# Optimizing vancomycin therapy: the critical role of dosing frequency, AUC monitoring and gender: a retrospective cohort study

**DOI:** 10.3389/fphar.2026.1843465

**Published:** 2026-08-19

**Authors:** Naiju Zhang, Yi Li, Longyun Chen, Zhenjie Wang, Tianping Chen

**Affiliations:** 1 Department of Pharmacy, Anhui Engineering Technology Research Center of Biochemical Pharmaceutical, National Clinical Research Center for Infectious Diseases, Institute of Emergency and Critical Care Medicine, First Affiliated Hospital of Bengbu Medical University, Bengbu, China; 2 Institute of Antibiotics, Huashan Hospital, and Key Laboratory of Clinical Pharmacology of Antibiotics, National Health Commission of People’s Republic of China, Fudan University, Shanghai, China; 3 Department of Emergency Surgery, Institute of Emergency and Critical Care Medicine, The First Affiliated Hospital of Bengbu Medical University, Bengbu, China; 4 Department of Cardiology, The First Affiliated Hospital of Bengbu Medical University, Bengbu, China

**Keywords:** AUC, dosing frequency, gender factors, nephrotoxicity, PK/PD, therapeutic drug monitoring, vancomycin

## Abstract

**Background:**

Optimizing vancomycin therapy requires balancing efficacy and toxicity. Although therapeutic drug monitoring (TDM) guided by area-under-the-curve (AUC) measurements is now recommended, the effect of dosing frequency and patient-specific factors remain underexplored. This study examined the relationships of dosing frequency, pharmacokinetic/pharmacodynamic (PK/PD) parameters, clinical efficacy, and toxicities in patients receiving different vancomycin regimens.

**Methods:**

This retrospective cohort study analyzed 193 vancomycin dosing regimens in 148 adult patients who were treated at a tertiary teaching hospital in China between January 2013 and March 2021, 175 of which were guided by TDM. The full TDM-guided cohort (n = 175) was used for analysis of safety and PK; clinical efficacy was assessed in 59 culture-confirmed cases; and the association of PK/PD parameters with efficacy was examined in the 26 patients who received a 1 g q12 h regimen for staphylococcal infections.

**Results:**

In the subset of 26 patients with staphylococcal infections who received the 1 g q12 h regimen, clinical success was not significantly associated with C_trough_, AUC, or AUC/minimum inhibitory concentration (AUC/MIC). For the entire TDM-guided cohort, dosing frequency was a significant and independent determinant of C_trough_ (P = 0.031), in that more frequent dosing led to higher C_trough_ values without altering total daily AUC. Receiver operating characteristic (ROC) analysis indicated that AUC was a better predictor of nephrotoxicity (AUROC = 0.754) than C_trough_ (AUROC = 0.659). Multivariate analysis confirmed that pre-treatment creatinine clearance was the primary predictor of post-treatment renal function. Female gender was an independent risk factor for post-treatment anemia (P = 0.002).

**Conclusion:**

Among the 26 patients with staphylococcal infections receiving the 1 g q12 h regimen, traditional PK/PD targets (C_trough_ and AUC/MIC) did not correlate with clinical efficacy in this real-world setting. AUC was a better predictor of nephrotoxicity than C_trough_, and female gender was an independent risk factor for vancomycin-associated anemia.

## Highlights

- The total dose and dosing frequency of vancomycin can affect patient outcome.
- The vancomycin AUC provided better predictions of nephrotoxicity than C_trough_.
- Females had a greater risk for vancomycin-associated anemia.
- Typical PK/PD targets (AUC, AUC/MIC, etc.) may unrelated to clinical efficacy.

## 1 Introduction

Vancomycin is a glycopeptide antibiotic that is primarily used to treat infections by Gram-positive bacteria, including methicillin-resistant *Staphylococcus aureus* (MRSA). The conventional recommended dose is 2 g per day, administered as 1 g every 12 h or 0.5 g every 6 h, with appropriate adjustments for age, body weight, and severity of illness (Chinese Expert Consensus, 2011). According to the 2011 Infectious Diseases Society of America (IDSA) guidelines for MRSA, the recommended vancomycin dosage is 15–20 mg/kg body weight every 8–12 h, with a maximum single dose of 2 g and a maximum daily dose generally not exceeding 4 g (Liu et al., 2011; Lodise et al., 2008). When administering high doses, the guidelines recommend monitoring of renal function (Lodise et al., 2008) and serum drug concentration. In addition, the vancomycin trough concentration (C_trough_) should be maintained above 10 μg/mL for common infections (e.g., uncomplicated skin or soft tissue infections and non-severe pneumonia) and between 15 and 20 μg/mL for complicated and severe infections by MRSA (Liu et al., 2011; Rybak et al., 2009).

Despite decades of clinical use, the vancomycin dosing strategy continues to evolve based on considerations of safety and efficacy. Although a target trough level of 15–20 μg/mL can provide clinical efficacy in patients with severe or complicated MRSA infections, this dose can also increase the risk of acute kidney injury (AKI). Consequently, balancing efficacy and safety is a central challenge for patients receiving vancomycin. This led to changes in the measurements used for therapeutic drug monitoring (TDM). The 2020 revised consensus guideline shifted focus to AUC-guided dosing, and recommend an AUC/minimum inhibitory concentration (MIC) ratio of 400–600 (assuming a microdilution MIC of 1 mg/L) as the primary pharmacokinetic/pharmacodynamic (PK/PD) target for serious MRSA infections, thus explicitly moving away from relying solely on C_trough_ targets (Rybak et al., 2020). This approach, which was also endorsed by other guidelines, emphasizes the need to optimize total drug exposure (AUC) to improve efficacy and renal safety (M et al., 2022; He et al., 2020).

However, dosing frequency is a critical aspect of vancomycin dosing that remains underexplored. Vancomycin exhibits time-dependent bactericidal activity with a moderate post-antibiotic effect, in that the time in which the serum concentration exceeds the MIC (T > MIC) is a key determinant of efficacy. The optimal bactericidal activity is achieved at concentrations 4- to 5-times greater than the MIC, and a higher peak concentration does not increase bacterial killing, consistent with a time-dependent killing pattern (Chinese Expert Consensus, 2011). Therefore, for the same total daily dose, a more fractionated regimen (e.g., 500 mg q6h) should theoretically increase the T > MIC compared to a regimen with a longer dosing interval (e.g., 1 g q12 h). A more fractionated daily dose could enhance bacterial killing without increasing peak concentration or cumulative exposure (AUC), and potentially provide a better balance of efficacy and safety. Despite the pharmacological plausibility of this approach, the clinical impact of dosing frequency on AUC, efficacy, and toxicity has not been systematically evaluated, particularly in diverse real-world populations. Furthermore, although the association between a higher vancomycin AUC and nephrotoxicity is well-established (Lodise et al., 2008; Rybak et al., 2020; Lodise et al., 2009; Bellos et al., 2020; van Hal et al., 2013; Aljefri et al., 2019; Tsutsuura et al., 2021), the predictive superiority of AUC over C_trough_ for this outcome is a cornerstone of current guidelines (Rybak et al., 2020; M et al., 2022; Aljefri et al., 2019; Tsutsuura et al., 2021; Avedissian et al., 2019; Neely et al., 2018). Meta-analyses have confirmed that AUC-guided monitoring is associated with a lower risk of AKI than C_trough_-guided monitoring (Aljefri et al., 2019; Tsutsuura et al., 2021; Abdelmessih et al., 2022). However, the relationship between these PK/PD targets and clinical efficacy in real-world settings remains complex, and there are inconsistencies in the results of previous studies (Tsutsuura et al., 2021; Lodise et al., 2020; Tongsai and Koomanachai, 2016; Wan et al., 2018; Kim et al., 2025; Fann et al., 2025; Limelette et al., 2025). For example, the PROVIDE trial (Lodise et al., 2020) and two meta-analyses (Tsutsuura et al., 2021; Tongsai and Koomanachai, 2016) found inconsistent associations between PK/PD targets and clinical success, suggesting that these targets are not sufficient in real-world settings.

Factors such as controlling the source of infection, host immune status, and pathogen virulence can confound the link between AUC and treatment success. Additionally, there is less known about other toxicities, such as hepatotoxicity and hematotoxicities (e.g., anemia), and the effects of patient-specific factors, such as gender, in modulating these risks.

This retrospective cohort study was designed to address these knowledge gaps. We aimed to systematically evaluate the effects of different vancomycin dosing regimens on the clinical outcomes and safety (nephrotoxicity, hepatotoxicity, and hematotoxicity/anemia) in a cohort of patients from a tertiary teaching hospital in China. Our primary objectives were to: (*i*) investigate the influence of dosing frequency, independent of total daily dose, on C_trough_ and toxicity; (*ii*) compare the predictive performance of AUC *vs.* C_trough_ for key toxicities; and (*iii*) identify the effect of patient-specific factors, such as gender, on vancomycin-related adverse events. The results of this study help to develop more personalized vancomycin therapies with optimization of dosing frequency based on AUC monitoring and patient-specific risk factors.

## 2 Patients and methods

### 2.1 Study design and ethical approval

This single-center, retrospective, observational cohort study was conducted at the First Affiliated Hospital of Bengbu Medical University (Bengbu, Anhui Province, China). The medical records of all patients who received intravenous vancomycin therapy from January 2013 to March 2021 were reviewed. The study protocol was approved by the local Institutional Ethics Committee (Protocol Code: 2020KY072; Date of Approval: 3 August 2020). Given the retrospective design, the Ethics Committee waived the requirement for written informed consent from patients. All study procedures were conducted in accordance with the Declaration of Helsinki.

### 2.2 Study population, data collection, and PK analysis

#### 2.2.1 Study population and data collection

**FIGURE 1:**
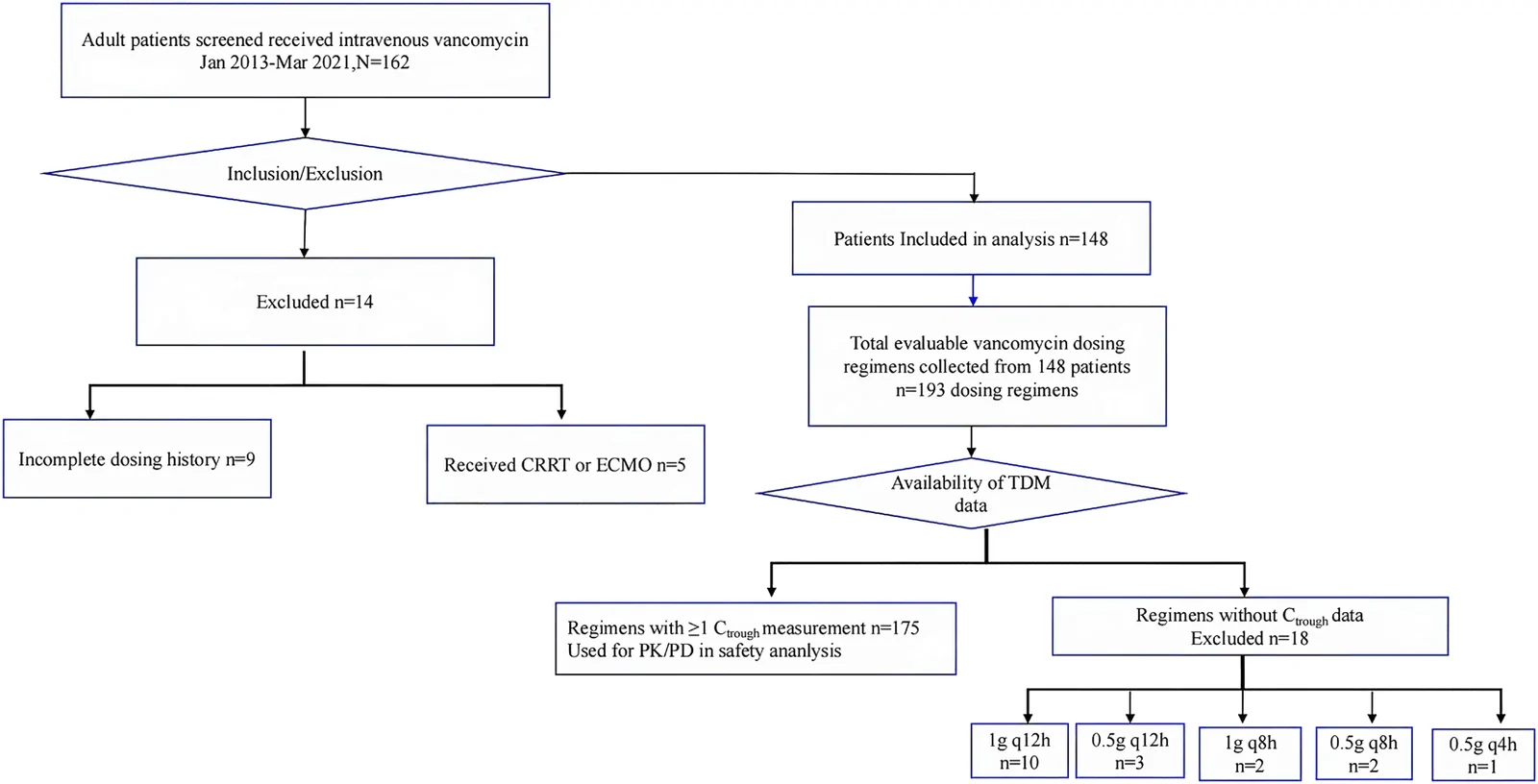
Procedures used to select patients (n = 148) and vancomycin dosing regimens (n = 193). CRRT, continuous renal replacement therapy; ECMO, extracorporeal membrane oxygenation; TDM, therapeutic drug monitoring.

All adult patients (age ≥18 years) who received intravenous vancomycin therapy for at least 72 h due to a confirmed or suspected infection by Gram-positive bacteria were included. Patients were excluded if their dosing history was incomplete, if they received continuous renal replacement therapy (CRRT) or extracorporeal membrane oxygenation (ECMO), or if their baseline laboratory data for safety analysis were missing (Figure 1). For patients with dose or frequency adjustments during therapy, each unique steady-state regimen (stable for ≥3 days before therapeutic drug monitoring) was treated as an independent regimen. Only steady-state regimens were included for the PK analysis. Clinical efficacy was only assessed in the 59 patients with culture confirmation. The full cohort was used for analysis of PK and safety.

Data extracted from the electronic medical records and the hospital’s TDM database included: (*i*) demographics (age, gender, weight); (*ii*) clinical data (admitting department, infection site, comorbidities, microbiological results including MIC); (*iii*) treatment details (vancomycin regimen, duration); (*iv*) TDM data (C_trough_, sample time); and (*v*) laboratory parameters measured pre- and post-treatment that were indicative of efficacy (e.g., body temperature, white blood cell count [WBC], C-reactive protein [CRP], procalcitonin [PCT]) and safety (serum creatinine [SCr], total bilirubin [TBIL], hemoglobin [Hb]).

#### 2.2.2 PK and laboratory analysis

Creatinine clearance (CrCl) was estimated using the Cockcroft-Gault formula (Cockcroft and Gault, 1976). Serum concentration was quantified using a homogeneous enzyme immunoassay (Emit® 2000 Vancomycin Assay, Siemens) on a Viva-E system (quantitative range: 2.0–50.0 μg/mL).

Individual PK parameters and the 24-h area under the curve (AUC_0 –24_) were estimated using JPKD software version 3.0 with maximum *a posteriori* Bayesian forecasting (Yu et al., 2022). Patient-specific data (demographics, SCr, dosing details, sampling times, and C_trough_) were entered into the software, which used an integrated population model to estimate individualized clearance (CL) and volume of distribution (Vd). The AUC_0 –24_ (µg⋅h/mL) was calculated as (total daily dose)/(individualized CL). The AUC/MIC was calculated using measured MIC values when available. The steady-state C_trough_ was measured using blood samples drawn 30 min before the 4th or 5th dose of the same dosing regimen. The medians of multiple measurements were reported, with exclusion of outliers (>3 IQR). In the 175 TDM-guided regimens, the median AUC was 423.43 (IQR 332.95–522.41) for single-measurement regimens (81.7%) and 412.09 (IQR 345.21–493.02) for those with ≥2 measurements (18.3%), indicating comparable estimates and acceptable robustness.

### 2.3 Definitions of outcomes

Clinical efficacy was assessed exclusively in the 59 patients with culture-confirmed infections by Gram-positive bacteria; patients who had suspected infections but were culture-negative were not included in any analysis of efficacy. Outcomes were classified as ‘clinical success’ (complete recovery or significant improvement) or ‘clinical failure’ (partial improvement or no effect) based on changes in symptoms, signs, and laboratory markers (WBC, CRP, PCT) after 72 h of treatment. These outcomes were adapted from established guidelines (The Clinical Pharmacology Base, 1987).

The three post-treatment toxicities (nephrotoxicity, hepatotoxicity, and hematotoxicity) were defined by specific laboratory criteria.

Nephrotoxicity was defined as a post-treatment CrCl below 50 mL/min based on the Cockcroft-Gault formula. For this analysis, we excluded 8 vancomycin regimens received by 7 patients in the original TDM-guided cohort (n = 175) who had baseline CrCl levels below 50 mL/min to avoid misclassification. This yielded 167 regimens (141 patients) for all nephrotoxicity-related evaluations (correlation, regression, and ROC curves). To address the limitation of using a fixed absolute threshold and to assess the robustness of our findings, we also performed sensitivity analyses using a ≥30% decline in CrCl or treating CrCl as a continuous variable. In these sensitivity analyses, for example, a patient with a pre-treatment CrCl of 130 mL/min and a post-treatment CrCl of 55 mL/min would be classified as nephrotoxic due to the ≥30% decline, even though the final CrCl was above 50 mL/min. For the primary definition, this threshold (post-treatment CrCl <50) was chosen because it represents a clinically relevant impairment of renal excretory function (consistent with moderate-to-severe chronic kidney disease). This study therefore assessed the overall safety outcome after treatment, rather than acute dynamic changes in SCr, which is typically required for diagnosis of AKI by the KDIGO criteria.

Hepatotoxicity was defined as a post-treatment TBIL level exceeding the upper limit of normal (ULN, >22 μmol/L), a standard threshold for abnormal hepatic excretory function. Anemia was defined as post-treatment Hb below 110 g/L (females) or below 120 g/L (males) as per our institutional laboratory criteria for descriptive and ROC analyses. In addition, post-treatment Hb was analyzed as a continuous variable in a multivariate model adjusting for baseline Hb, while absolute Hb change (post-treatment minus baseline) was examined separately.

### 2.4 Statistical analysis

Statistical analysis was performed using SPSS version 25.0 (IBM Corp., United States). A two-sided P-value below 0.05 was considered statistically significant. Normality was assessed using the Shapiro-Wilk test. Continuous variables are presented as mean ± standard deviation (SD) or median with interquartile range (IQR), as appropriate. Categorical variables are expressed as counts and percentages (n, %).

Group comparisons were performed using the Mann-Whitney U test (two groups) or the Kruskal–Wallis H test (multiple groups). Within-group comparisons (pre-treatment *vs.* post-treatment) used the Wilcoxon signed-rank test. Correlations were assessed using Spearman’s rank correlation coefficient.

Variables with a *P* below 0.05 in univariate analyses were considered for inclusion in the multivariable models. However, the final models were fitted using the forced entry (Enter) method, in which all prespecified clinically relevant covariates (age, sex, body weight, baseline laboratory values, vancomycin exposure metrics etc.) were entered simultaneously. Stepwise selection was not used. Sex was included *a priori* based on clinical plausibility, regardless of its univariate P-value. All results are presented as adjusted coefficients (β) with 95% confidence intervals (CIs).

Receiver operating characteristic (ROC) curve analysis was performed to assess the predictive performance of C_trough_ and AUC for the three post-treatment toxicities, with calculation of area under the ROC curve (AUROC) and the Youden index as the optimal cutoff.

## 3 Results

### 3.1 Patient characteristics

We identified 148 adult patients who received 193 evaluable vancomycin dosing regimens at our institution between January 2013 and March 2021 (Table 1). The cohort had a median age of 55 years (IQR: 46–68), a median weight of 60.5 kg (IQR: 54–72), and 65.5% (97/148) were male. The most common sites of infections were the lungs (41.9%) and bloodstream (25.7%). The median treatment duration was 8 days (IQR 5–14), with no difference between the success and failure groups. Sex-stratified safety data are presented in Table 2. Concomitant use of 2–3 nephrotoxic agents occurred in 28.48% (55/193) of all regimens (Supplementary Table S1).

**TABLE 1:** Demographic and clinical characteristics of enrolled patients (n = 148).

Characteristic	Number (%)
Year of treatment	
2014	2 (1.35)
2015	2 (1.35)
2016	4 (2.70)
2017	10 (6.76)
2018	16 (10.81)
2019	55 (37.16)
2020	51 (34.46)
2021	8 (5.41)
Admitting department	
Infectious diseases	41 (27.71)
Medical oncology	2 (1.35)
Cardiac surgery	6 (4.05)
NSICU	4 (2.70)
Emergency internal medicine	6 (4.05)
ICU	13 (8.78)
RICU	40 (27.03)
Pneumology	10 (6.76)
Neurosurgery	14 (9.46)
Hematology	1 (0.67)
Dermatology	1 (0.67)
Cardiovascular	1 (0.67)
Vascular surgery	1 (0.67)
Otolaryngology	1 (0.67)
Rehabilitation	4 (2.70)
Rheumatology and immunology	2 (1.35)
Ophthalmology	1 (0.67)
Demographics	
Gender, male/female	97/51
Age, years [median (range)]	55 (46–68)
Weight, kg [median (range)]	60.5 (54–72)
Co-existing or underlying disease	
None	88 (59.46)
Cardiovascular disease	12 (8.11)
Liver disorder	10 (6.76)
Renal disorder	2 (1.35)
Cancer	5 (3.37)
Rheumatic and autoimmune disease	6 (4.05)
Endocrine system disease	5 (3.38)
Digestive system disease	3 (2.03)
Central nervous system disease	12 (8.11)
Hematological system diseases	4 (2.70)
Diseases of urinary system	2 (1.35)
Oculopathy	3 (2.03)
Respiratory disease	5 (3.38)
Site of infection	
Pulmonary	62 (41.89)
Bloodstream	36 (24.32)
Intraocular	3 (2.03)
Chest	18 (12.16)
Skin	4 (2.70)
Urinary tract	7 (4.73)
Central nervous system	14 (9.46)
Infectious endocarditis	10 (6.76)
Sample used for culture	
Blood	22 (50.00)
Excretion	6 (13.64)
Sputum	5 (11.36)
Hydrothorax	4 (9.09)
Bone marrow	4 (9.09)
CSF	4 (9.09)
Throat swab	1 (2.27)
Aqueous humor	1 (2.27)
In-dwelling catheter	1 (2.27)

NSICU, neurosurgical intensive care unit; ICU, intensive care unit; RICU, respiratory intensive care unit; CSF, cerebrospinal fluid.

**TABLE 2:** Changes in renal, hematological, and hepatic parameters by sex*.

Sex	n	Median ΔCrCl	Z	P	Median ΔHb	Z	P	Median ΔTBil	Z	P
Male	97	8.85 (−8.3 to +17.7)	−1.228	0.219	−3.0 (−13.0 to +4.0)	−0.221	0.825	−1.3 (−6.3 to +1.1)	−0.504	0.614
Female	51	−1.34 (−16.1 to +20.8)			−3.0 (−11.25 to +7.5)			−2.7 (−4.9 to +0.5)		

* Data shown as median (IQR). ΔCrCl, change in creatinine clearance (mL/min); ΔHb, change in hemoglobin (g/L); ΔTBil, change in total bilirubin (μmol/L). Negative values indicate a decrease from baseline. P values were calculated using the Mann–Whitney U test.

**FIGURE 2:**
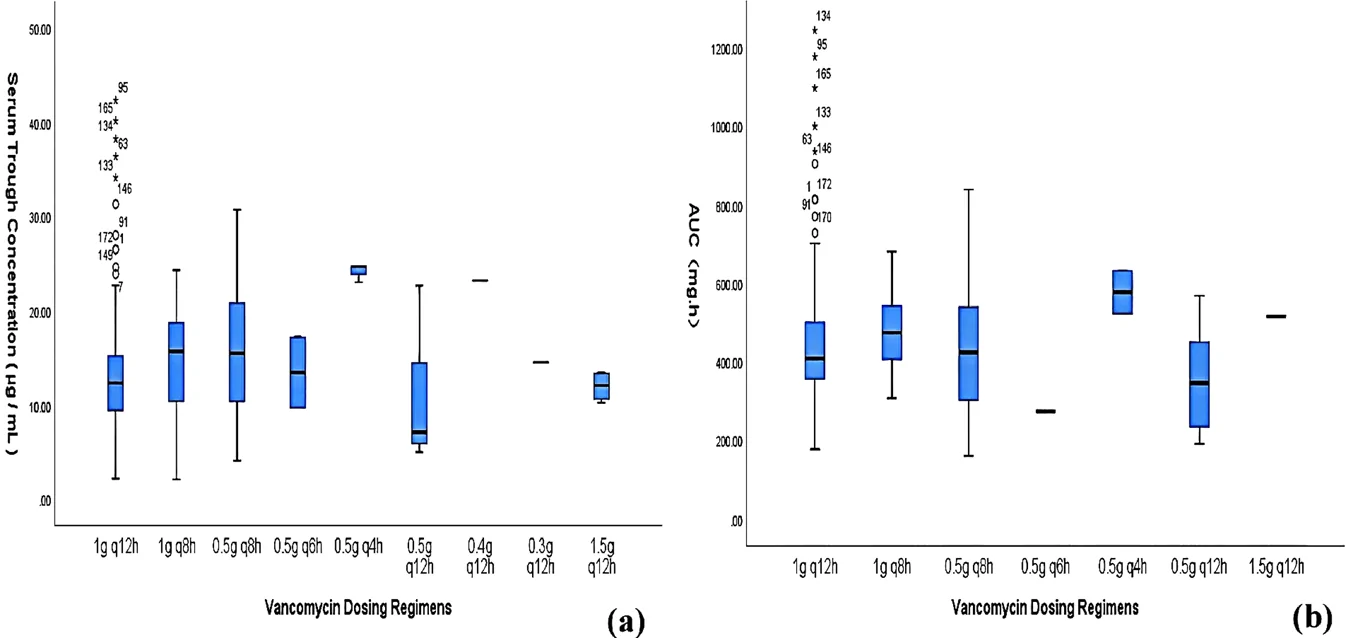
Distributions of C_trough_ **(a)** and AUC **(b)** for regimens that used TDM (n = 175). Boxes represent median and IQR; whiskers represent range.

We then analyzed the PK/PD and safety of the 175 evaluable regimens (90.7%; 217 total measurements) that used TDM (Figure 2). Of the 175 TDM-guided regimens, 73 (41.7%) were administered during the COVID-19 pandemic period (2020–2021), while the remaining 102 (58.3%) were from the pre-pandemic era (2013–2019). Baseline characteristics stratified by period are shown in Supplementary Table S2. The most common regimen was 1 g q12 h (n = 120). The remaining regimens included 1 g q8 h (n = 15), 0.5 g q8 h (n = 21), 0.5 g q12 h (n = 8), 1.5 g q12 h (n = 4), 0.5 g q6 h (n = 2), and 0.5 g q4 h (n = 5) (Table 5). For all evaluable regimens, the median C_trough_ was 12.6 μg/mL (IQR: 9.5–17.5) and the median AUC was 417.2 µg⋅h/mL (IQR: 345.0–521.7).

Of the 59 culture-confirmed cases, 54 were also included in the 175 TDM-guided regimens and thus had complete AUC and C_trough_ measurements. These 54 regimens constituted the analytical cohort for the pandemic-stratified efficacy analysis; the remaining 5 culture-confirmed cases lacked TDM data and were excluded from this specific analysis.

**FIGURE 3:**
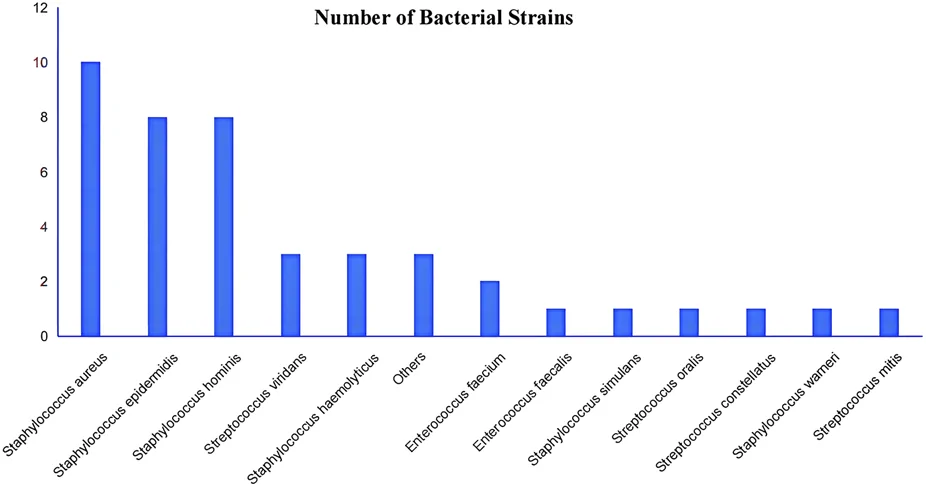
The distribution of bacterial strains (n = 59).

Among patients with available culture data, 5.08% (3/59) had multiple Gram-positive isolates from different sites. Figure 3 shows the numbers of the 13 bacterial strains.

### 3.2 Clinical efficacy in *Staphylococcus* infections is not correlated with traditional PK/PD targets (C_trough_, AUC, AUC/MIC)

Among the 59 culture-confirmed Gram-positive cases, 51 (86.4%) achieved clinical success. Of the 26 patients with staphylococcal infections who received the 1 g q12 h regimen, the species distribution was as follows: MRSA in 6 patients (23.1%), MSSA in 3 patients (11.5%), and coagulase-negative staphylococci (CoNS) in 19 patients (73.1%) (Supplementary Table S3). The median vancomycin MIC was 1 μg/mL (IQR: 0.875–1.0, by broth microdilution); the AUC of 345 μg h/mL indicated the AUC/MIC was below 400, which is suboptimal. The MIC90 was not routinely reported. These 26 patients experienced significant decreases in body temperature, white blood cell count, neutrophil percentage, and C-reactive protein (all P < 0.05), thereby confirming clinical efficacy (Table 3). However, a comparison of patients in this subgroup who achieved clinical success (n = 22) *vs.* failure (n = 4) indicated no significant differences in median C_trough_ (13.0 *vs.* 8.3 μg/mL, P = 0.604), AUC (417.2 *vs.* 363.7 µg⋅h/mL, P = 0.853), or AUC/MIC (459.1 *vs.* 363.7, P = 0.387) (Table 4). This suggests that although vancomycin was effective, achieving a specific PK/PD target should not be considered the sole determinant of clinical success in this real-world setting. Given the small number of failures (n = 4), this subgroup analysis was underpowered, so these results should be interpreted with caution. Based on the difference in AUC between the success and failure groups (median: 417.19 *vs.* 363.72 µg⋅h/mL), *post hoc* power analysis revealed that the achieved power was approximately 19.3%; for C_trough_ (median: 13.00 *vs.* 8.25 μg/mL), the achieved power was approximately 50.8% (α = 0.05, two-sided). Both of these values were below the conventional 80% threshold. To address potential confounding by concomitant antibiotics, we also examined the use of anti-staphylococcal agents in this subgroup (Supplementary Table S4). Microbiologic follow-up data are presented in Supplementary Table S5.

**TABLE 3:** Clinical efficacy of the most common vancomycin dosing regimen (1g q12 h) in patients with confirmed staphylococcal infections (n = 26).

Indicator	Pre-administration	Post-administration	Z	P
T_max_, °C	38.85 (37.60, 39.63)	37.10 (36.58, 37.80)	−4.172	<0.001
WBC, 10^9^/L	10.75 (6.90, 13.67)	7.96 (4.91, 10.37)	−2.128	0.033
N, %	80.55 (75.00, 85.88)	70.30 (62.18, 76.73)	−2.763	0.006
CRP, mg/L	90.00 (51.25, 198.00)	17.00 (7.73, 45.65)	−3.622	<0.001
PCT, ng/mL	0.50 (0.11, 3.39)	0.15 (0.1, 0.34)	−1.761	0.078

T_max_, maximal body temperature; WBC, white blood cells; N, neutrophils; CRP, C reactive protein; PCT, procalcitonin.

Values indicate median (IQR).

**TABLE 4:** Relationship of clinical outcome with PK/PD parameters in patients with staphylococcal infections who received the 1 g q12 h vancomycin regimen (n = 26).

Parameter	Clinical outcome		*Z*	*P*
	Success (n = 22)	Failure (n = 4)		
C_trough_, µg/mL	13.00 (9.60, 15.45)	8.25 (5.88, 13.48)	0.764	0.604
AUC, µg⋅h/mL	417.19 (360.33, 542.30)	363.72 (268.57, 444.68)	0.609	0.853
AUC/MIC	459.13 (271.24, 826.75)	363.72 (268.57, 444.68)	0.905	0.387

Values indicate median (IQR).

### 3.3 Dosing frequency is independently associated with c_trough_

**FIGURE 4:**
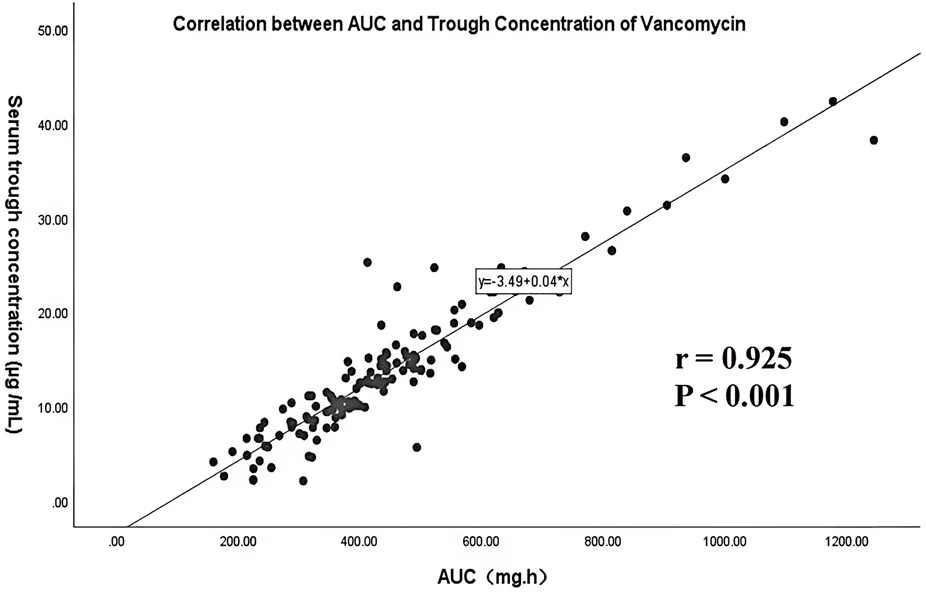
Correlation between C_trough_ and AUC for regimens that used TDM (n = 175). Spearman’s correlation coefficient (r) and P-value are shown.

The total daily dose varied among the seven most common dosing regimens and there was a relationship between dosing frequency and AUC. In particular, regimens with comparable total daily doses but different dosing frequencies had significant differences in C_trough_ (P = 0.031, Kruskal–Wallis test) but not in AUC (P = 0.146) (Table 5, Figure 2). Regimens with more frequent dosing (e.g., 0.5 g q4 h, total daily dose 3 g) tended to have a higher C_trough_ than regimens with less frequent dosing but a similar total daily dose. Multivariate linear regression analysis (Table 6) confirmed that dosing frequency was an independent predictor of C_trough_, even after adjusting for body weight and AUC. Two specific comparisons (1.5 g q12 h *vs.* 0.5 g q4 h, β = −7.849, P = 0.001; and 1 g q8h *vs.* 0.5 g q4h, β = −5.977, P < 0.001), demonstrated that more frequent dosing was associated with a significantly greater C_trough_ for a given total daily exposure (AUC_0–24_). As expected, C_trough_ and AUC had a strong positive correlation (r = 0.925, P < 0.001; Figure 4).

**TABLE 5:** PK/PD parameters in the seven most common dosing regimens that used TDM (**n = 175**). Values indicate median (IQR).

Parameter	1 g q12 h (n = 120)	1 g q8 h (n = 15)	0.5 g q8 h (n = 21)	0.5 g q12 h (n = 8)	1.5 g q12 h (n = 4)	0.5 g q6 h (n = 2)	0.5 g q4 h (n = 5)	H	P
C_trough_ (μg/mL)	12.35 (9.43, 15.25)	15.70 (7.75, 18.85)	15.50 (10.19, 21.40)	7.15 (5.55, 14.58)	12.10 (10.44,13.43)	13.45 (7.28, 14.55)	24.70 (23.00, 24.70)	13.863	0.031
AUC (µg⋅h/mL)	407.80 (354.19, 508.21)	473.48 (376.41, 582.21)	423.44 (294.37, 546.71)	345.21 (232.61, 460.82)	*ND	*ND	576.50 (391.30, 477.75)	8.193	0.146

* ND: no data.

**TABLE 6:** Multivariate analysis of the association of different factors with C_trough_ (n = 175) and vancomycin-associated adverse events (CrCl post-treatment, n = 167; TbiL post-treatment, n = 175; Hb post-treatment, n = 175) in regimens that used TDM.

Variable	Factor	B	Std. Error	95% wald CI		Hypothesis test	
				Lower	Upper	Wald χ^2^	P
C_trough_	(Intercept)	−11.732	2.2149	−16.073	−7.391	28.060	<0.001
	0.5 g q8 h *vs.* 1 g q12 h	*ND	0.473	2.271	4.125	*ND	<0.001
	1 g q12 h *vs.* 0.5 g q4 h	*ND	1.360	−8.580	−3.248	*ND	<0.001
	0.5 g q12 h *vs.* 1 g q12 h	*ND	0.9166	2.138	5.731	*ND	<0.001
	1 g q8 h *vs.* 0.5 g q8 h	*ND	0.6877	−4.609	−1.913	*ND	<0.001
	1.5 g q12 h *vs.* 0.5 g q4 h	*ND	2.2880	−12.334	−3.365	*ND	0.001
	1.5 g q12 h *vs.* 0.5 g q12 h	*ND	2.0821	−9.951	−1.789	*ND	0.005
	1 g q8 h *vs.* 0.5 g q4 h	*ND	1.4059	−8.733	−3.222	*ND	<0.001
	1 g q8 h *vs.* 0.5 g q12 h	*ND	1.0545	−6.065	−1.931	*ND	<0.001
	1.5 g q12 h *vs.* 0.5 g q8 h	*ND	1.9420	−8.939	−1.327	*ND	0.008
	0.5 g q8 h *vs.* 1 g q12 h	*ND	0.473	2.271	4.125	*ND	<0.001
	Age (years)	0.017	0.0136	−0.010	0.044	1.570	0.210
	Body weight (kg)	0.055	0.0151	0.025	0.084	13.328	<0.001
	Pre-treatment CrCl (mL/min)	0.005	0.0057	−0.006	0.016	0.755	0.385
	AUC (mg.h)	0.040	0.0010	0.038	0.042	1738.758	<0.001
CrCl post-treatment	(Intercept)	89.321	35.6497	19.449	159.193	6.278	0.012
	Daily dose(g)	12.076	10.0864	−7.693	31.845	1.433	0.231
	Gender (M/F)	15.571	8.3817	−0.857	31.999	3.451	0.063
	Age (years)	−0.436	0.3090	−1.042	0.169	1.993	0.158
	Body weight (kg)	0.601	0.3272	−0.040	1.242	3.374	0.066
	AUC (mg.h)	−0.143	0.0834	−0.307	0.020	2.944	0.086
	C_trough_	0.861	1.8627	−2.790	4.512	0.214	0.644
	Pre-treatment CrCl (mL/min)	0.240	0.1173	0.010	0.470	4.191	0.041
TbiL post-treatment	(Intercept)	−18.033	8.2725	−34.247	−1.819	4.752	0.029
	Body weight (kg)	0.492	0.1290	0.239	0.745	14.530	<0.001
Hb post-treatment	(Intercept)	92.619	11.2417	70.586	114.653	67.880	<0.001
	Gender (M/F)	10.735	3.5048	3.866	17.604	9.381	0.002
	Body weight (kg)	0.034	0.1269	−0.215	0.283	0.071	0.789
	AUC (mg^.^h)	0.001	0.0295	−0.057	0.058	<0.001	0.985
	C_trough_	−0.709	0.6908	−2.063	0.645	1.053	0.305
	Daily dose	6.519	3.7060	−0.744	13.783	3.094	0.079

* ND: no data.

For the CrCl post-treatment model, 8 regimens from patients with baseline CrCl <50 mL/min were excluded (n = 167).

### 3.4 AUC is a better predictor of nephrotoxicity than C_trough_

**FIGURE 5:**
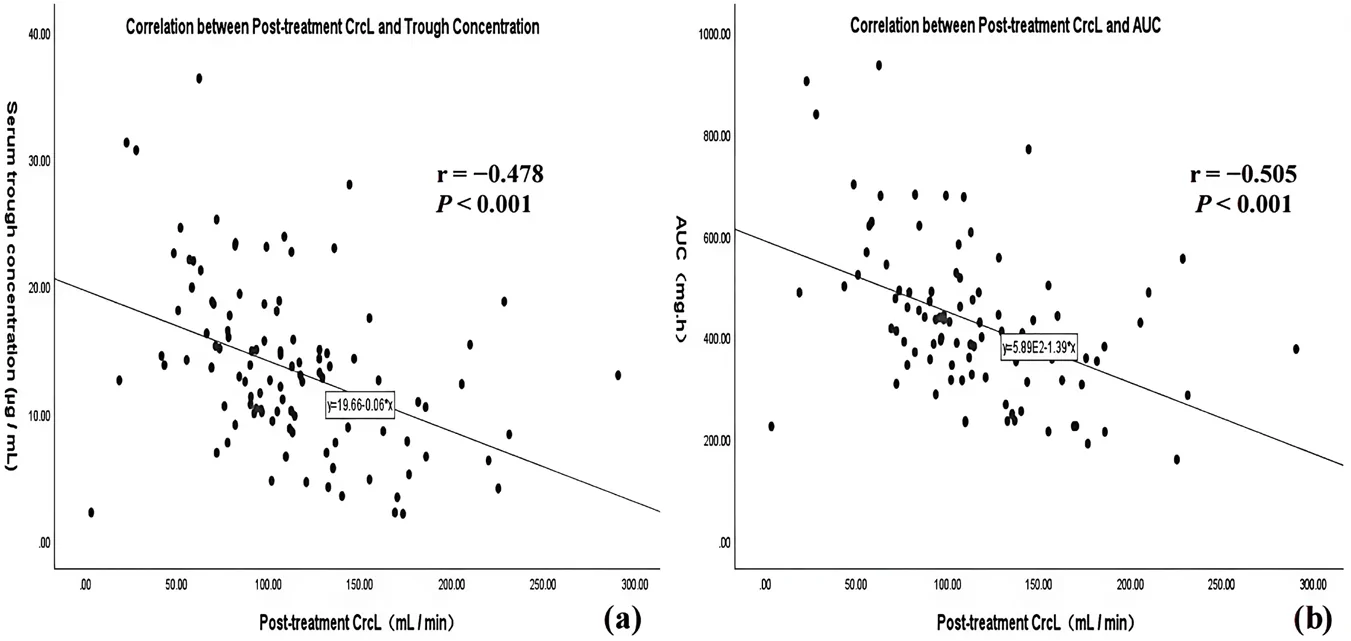
Correlation of post-treatment CrCl with C_trough_ **(a)** and AUC **(b)** for regimens that used TDM (n = 167). Spearman’s correlation coefficients (r) and P-values are shown. CrCl, creatinine clearance.

**FIGURE 6:**
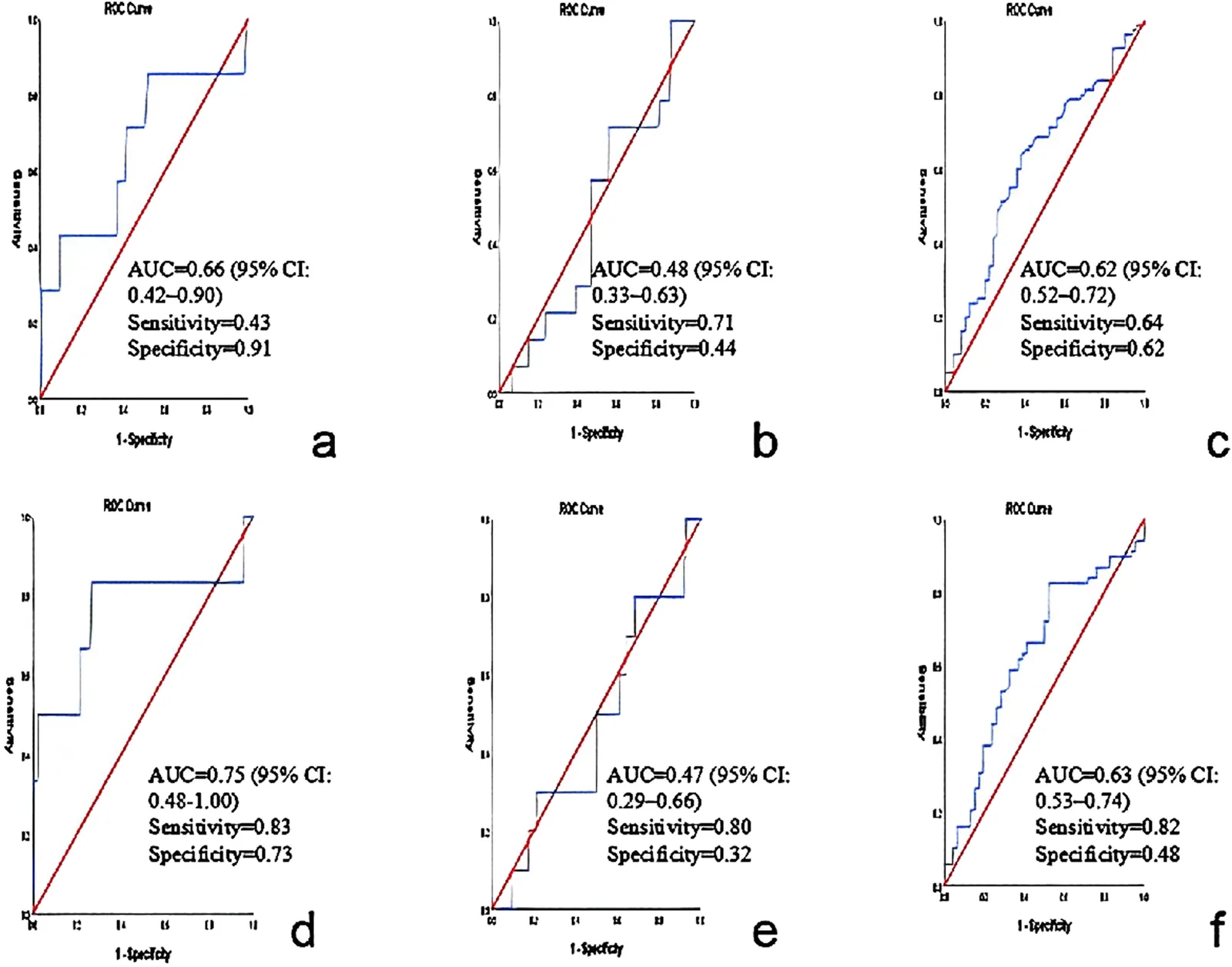
ROC curves for C_trough_ (top row) and AUC (bottom row) in predicting nephrotoxicity **(a,d)**; CrCl < 50 mL/min, n = 167), hepatotoxicity **(b,e)**; TBIL > 22 μmol/L, n = 175), and hematotoxicity **(c,f)**; Hb < 110 g/L, n = 175) for regimens that used TDM. AUROC (area under the ROC curve) and 95% confidence intervals are shown. ROC, receiver operating characteristic; CrCl, creatinine clearance; TBIL, total bilirubin; Hb, hemoglobin.

Among the 175 TDM-guided regimens, 8 regimens from 7 patients with baseline CrCl below 50 mL/min were excluded from the nephrotoxicity analysis (See Patients and Methods), leaving 167 regimens (141 patients) for evaluation. Among these 167 regimens with baseline CrCl of at least 50 mL/min, 7 (4.2%) developed post-treatment CrCl below 50 mL/min. Both C_trough_ and AUC had significant negative correlations with post-treatment CrCl ((r = −0.478, P < 0.001 and r = −0.505, P < 0.001, respectively; Table 7, Figure 5). When the relative change in renal function was assessed using the percent decline in CrCl (Δ%), both C_trough_ and AUC also showed weak but statistically significant negative correlations (r = −0.252, P = 0.011 and r = −0.264, P = 0.009, respectively; Table 7), indicating that higher vancomycin exposure was associated with a greater relative decline in renal function. ROC analysis confirmed that both metrics successfully predicted nephrotoxicity (CrCl <50 mL/min), but AUC had superior performance (Figure 6). In particular, for C_trough_, the AUROC was 0.659 (95% CI: 0.415–0.903), with an optimal cutoff was 22.35 μg/mL, a sensitivity of 42.86%, and a specificity of 90.57% (Figure 6a); for AUC, the AUROC was 0.754 (95% CI: 0.484-1.000), with an optimal cutoff of 481.99 µg⋅h/mL, a sensitivity of 83.3%, and a specificity of 73.1% (Figure 6d). To assess the robustness of our primary findings, we performed sensitivity analyses defining nephrotoxicity as a ≥30% decline in CrCl or treating CrCl as a continuous variable (Table 7). In the ≥30% decline analysis, the AUROC for AUC was 0.629 (95% CI: 0.396–0.863) and for C_trough_ was 0.615 (95% CI: 0.426–0.804).

**TABLE 7:** Correlation of C_trough_ and AUC with post-treatment nephrotoxicity, hepatotoxicity, and anemia/hematotoxicity for regimens that used TDM (n = 175).

Indicator	C_trough_ (r, *P*)	AUC (r, *P*)
Post-treatment CrCl (mL/min)	−0.478, <0.001	−0.505, <0.001
Δ%	−0.252, 0.011	−0.264, 0.009
Post-treatment TBIL (µmol/L)	−0.097, 0.289	−0.072, 0.456
Post-treatment hb (g/L)	−0.278, 0.001	−0.268, 0.004

CrCl, creatinine clearance; Hb, hemoglobin; TBIL, total bilirubin.

For the CrCl post-treatment model, 8 regimens from patients with baseline CrCl <50 mL/min were excluded (n = 167).

Δ% = (Post-treatment CrCl–Pre-treatment CrCl)/Pre-treatment CrCl × 100%.

In contrast to the predictive analysis, multivariate analysis of factors associated with post-treatment CrCl demonstrated that pre-treatment CrCl was the only significant independent predictor (β = 0.240, P = 0.041). The effects of AUC (P = 0.086) and C_trough_ (P = 0.644) were not significant, likely due to their strong correlation with pre-treatment renal function and the limited number of nephrotoxicity events (Table 6).

To formally evaluate whether the pandemic period modified the association between vancomycin AUC and nephrotoxicity, we tested an AUC × Pandemic interaction term in the multivariable logistic regression model. The interaction term was not statistically significant (β = −0.0088, P = 0.094), and the overall model was significant (Omnibus χ^2^ = 11.036, df = 2, P = 0.004). This indicates that the association between vancomycin AUC and nephrotoxicity remained consistent across the pre-pandemic and pandemic periods, further supporting the robustness of our main findings despite the baseline differences between cohorts.

### 3.5 Female gender is an independent risk factor for vancomycin-associated anemia

**FIGURE 7:**
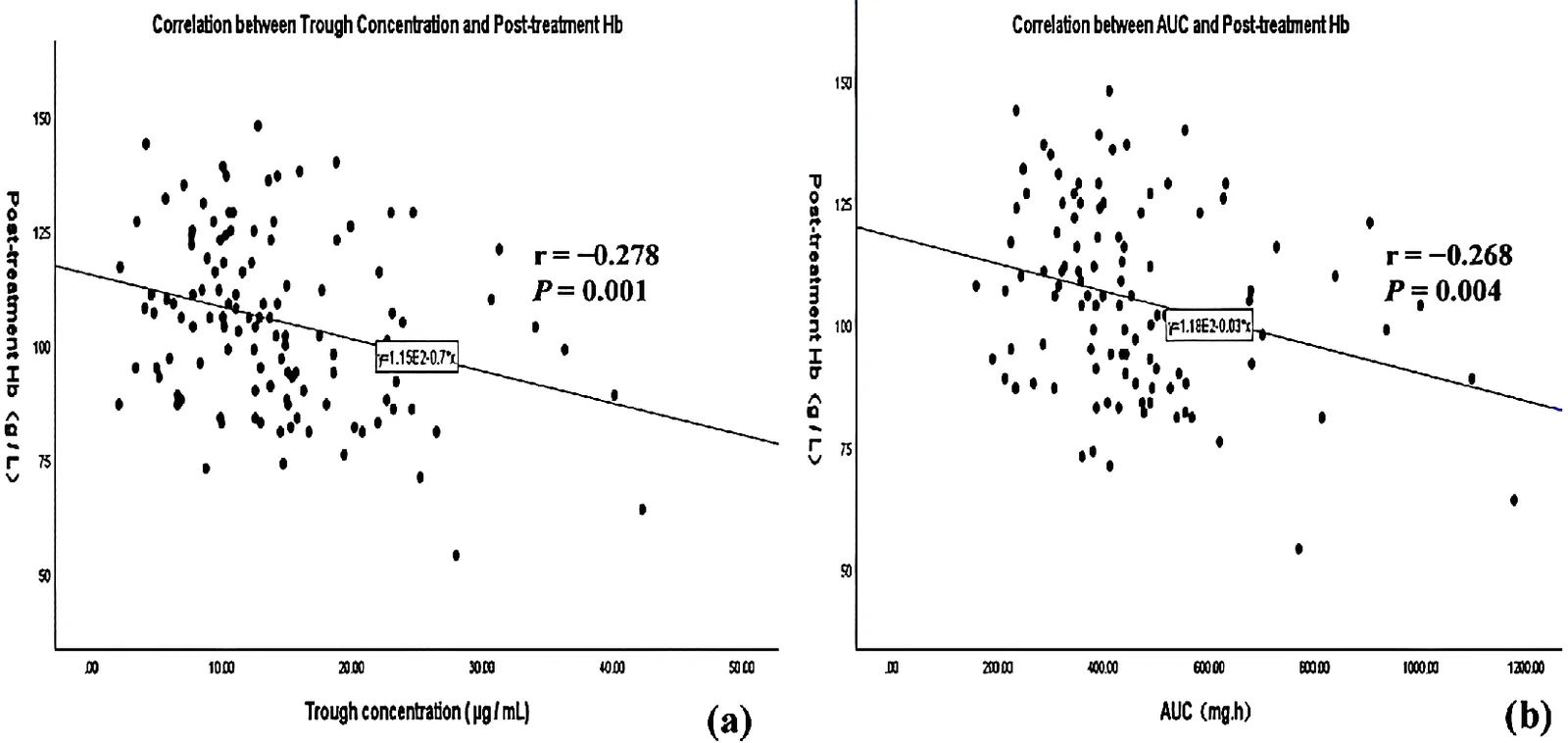
Correlation of post-treatment Hb level with C_trough_ **(a)** and AUC **(b)** for regimens that used TDM (n = 175). Spearman’s correlation coefficients (r) and P-values are shown. Hb, hemoglobin.

There were significant differences in the hematological parameters among the different regimens (Table 8). Notably, the 1 g q8 h and 0.5 g q8 h regimens were associated with significant decreases in post-treatment Hb (P = 0.039 and P = 0.041, respectively). Both C_trough_ and AUC had weak but significant negative correlations with the post-treatment Hb (r = −0.278, P = 0.001 and r = −0.268, P = 0.004, respectively; Table 7, Figure 7). However, ROC analysis showed that neither metric was a strong predictor of anemia (AUROC = 0.62 for C_trough_, Figure 6c; AUROC = 0.63 for AUC, Figure 6f).

**TABLE 8:** Changes of laboratory safety indicators in the four most common dosing regimens (n = 193)**.** Values indicate median (IQR).

Regimen	Indicator	Pre-administration	Post-administration	Z	*P*
1g q12 h (n = 121)	RBC (×10^12^/L)	3.70 (3.26–4.25)	3.57 (3.12–4.02)	−1.925	0.054
	Hb (g/L)	110 (96.00–126.00)	106.00 (91.00–122.00)	−1.815	0.070
	PLT (×10^9^/L)	257.00 (183.50–355.50)	255.00 (192.00–376.50)	−0.486	0.627
	ALT (U/L)	34.00 (17.00–53.25)	30.00 (19.00–55.75)	−0.275	0.783
	AST (U/L)	31.00 (20.00–49.50)	25.50 (20.25–54.00)	−0.998	0.318
	ALP (U/L)	86.50 (66.50–127.00)	90.50 (66.00–121.00)	−0.123	0.902
	r-GT (U/L)	48.00 (25.00–103.75)	49.50 (31.00–123.25)	−1.549	0.121
	TBIL (µmol/L)	9.85 (6.58–16.63)	7.30 (5.13–11.85)	−3.252	0.001
	DBIL (µmol/L)	3.40 (1.80–6.20)	2.75 (1.83–5.55)	−1.859	0.063
	Scr (µmol/L)	64.00 (54.00–71.00)	62.50 (49.00–72.00)	−1.608	0.108
	CrCl (mL/min)	94.01 (77.35–120.70)	106.56 (72.57–134.33)	−1.773	0.076
	BUN (mmol/L)	4.97 (3.51–6.76)	4.72 (3.08–6.24)	−1.918	0.055
1g q8 h (n = 17)	RBC (×10^12^/L)	3.54 (3.48–4.08)	3.48 (3.26–4.41)	−0.804	0.422
	Hb (g/L)	107.00 (99.50–124.50)	102.00 (93.00–124.00)	−2.065	0.039
	PLT (×10^9^/L)	340.00 (247.00–417.00)	279.00 (227.50–432.50)	−0.874	0.382
	ALT (U/L)	27.00 (18.50–60.00)	24.00 (17.00–52.25)	−1.664	0.096
	AST (U/L)	31.00 (20.50–44.00)	26.50 (18.00–35.50)	−0.979	0.328
	ALP (U/L)	90.00 (67.00–110.00)	93.50 (56.75–123.00)	−0.911	0.362
	r-GT (U/L)	54.00 (23.50–98.00)	46.00 (18.25–79.50)	−2.072	0.038
	TBIL (µmol/L)	7.90 (6.15–10.90)	6.30 (4.83–7.93)	−1.633	0.103
	DBIL (µmol/L)	2.35 (1.38–3.50)	2.20 (1.63–2.60)	−0.706	0.480
	Scr (µmol/L)	59.00 (50.00–65.50)	58.00 (52.00–62.50)	−0.668	0.504
	CrCl (mL/min)	114.19 (95.15–131.53)	113.52 (98.34–141.39)	−0.445	0.657
	BUN (mmol/L)	4.74 (2.95–6.80)	4.60 (3.94–6.64)	−0.314	0.754
0.5g q8 h (n = 22)	RBC (×10^12^/L)	3.78 (3.25–4.28)	3.60 (2.74–4.04)	−1.922	0.055
	Hb (g/L)	112.00 (98.00–124.50)	108.00 (81.50–112.50)	−2.043	0.041
	PLT (×10^9^/L)	319.00 (160.50–367.00)	231.00 (151.50–375.50)	−0.735	0.463
	ALT (U/L)	17.00 (12.75–33.75)	24.00 (14.00–43.00)	−0.105	0.916
	AST (U/L)	25.00 (19.00–34.75)	23.00 (18.00–42.00)	−0.392	0.695
	ALP (U/L)	90.00 (76.50–142.50)	93.00 (69.00–131.00)	−2.261	0.024
	r-GT (U/L)	37.00 (28.75–128.75)	53.00 (30.00–91.00)	−2.354	0.019
	TBIL (µmol/L)	10.00 (7.43–13.03)	6.90 (5.00–9.70)	−2.668	0.008
	DBIL (µmol/L)	2.95 (2.10–3.60)	2.70 (1.60–5.20)	−1.125	0.260
	Scr (µmol/L)	63.00 (47.75–73.25)	63.00 (49.00–82.00)	−0.346	0.730
	CrCl (mL/min)	83.31 (61.70–128.76)	78.87 (59.05–136.52)	−0.973	0.331
	BUN (mmol/L)	5.40 (3.60–7.06)	4.84 (3.11–6.12)	−1.538	0.124
0.5g q12 h (n = 11)	RBC (×10^12^/L)	2.99 (2.84–3.11)	3.01 (2.92–3.67)	−0.297	0.767
	Hb (g/L)	88.00 (86.00–101.00)	93.00 (87.00–98.00)	−0.534	0.593
	PLT (×10^9^/L)	147.00 (128.00–315.00)	246.00 (162.00–563.00)	−1.719	0.086
	ALT (U/L)	17.50 (11.00–29.50)	12.00 (11.00–52.00)	−1.693	0.090
	AST (U/L)	18.00 (16.00–34.00)	18.50 (17.75–50.00)	−1.696	0.090
	ALP (U/L)	84.50 (52.00–123.25)	105.00 (67.00–192.25)	−0.280	0.779
	r-GT (U/L)	34.00 (17.00–67.25)	63.50 (25.75–194.50)	−1.690	0.091
	TBIL (µmol/L)	6.35 (4.45–13.60)	7.20 (4.78–19.93)	−0.491	0.624
	DBIL (µmol/L)	2.70 (2.00–4.80)	2.40 (1.53–3.15)	−0.135	0.893
	Scr (µmol/L)	46.5 (35.50–84.25)	41.00 (37.00–66.50)	−0.280	0.779
	CrCl (mL/min)	81.83 (46.48–90.47)	79.78 (55.56–109.53)	−0.169	0.866
	BUN (mmol/L)	5.17 (3.48–14.19)	5.68 (3.62–8.96)	−0.140	0.889

RBC, red blood cell count; Hb, hemoglobin; PLT, platelets; ALT, alanine aminotransferase; AST, aspartate aminotransferase; ALP, alkaline phosphatase; r-GT, gamma-glutamyl transferase; TBIL, total bilirubin; DBIL, direct bilirubin; Scr, serum creatinine; CrCl, creatinine clearance; BUN, blood urea nitrogen.

Crucially, multivariate analysis (Table 5) that used post-treatment Hb as a continuous variable and adjusted for baseline Hb indicated that female gender was a strong and independent risk factor for a lower post-treatment level of Hb (β = 10.735, P = 0.002). Body weight, AUC, C_trough_, and daily dose were not significant in this model. To further validate this finding, we directly compared the intra-individual change in hemoglobin (ΔHb) between sexes; although this univariate comparison did not show a statistically significant difference (P = 0.825) (Table 2), the multivariate-adjusted effect remained robust, confirming that the impact of gender on Hb decline is independent of baseline differences (Table 5).

To evaluate whether the pandemic period modified the association between sex and post-treatment hemoglobin (Hb), we performed stratified analyses adjusting only for baseline Hb. In the pre-pandemic cohort, female sex was not significantly associated with post-treatment Hb (β = −1.105, P = 0.740). In the pandemic cohort, the association remained non-significant (β = −2.183, P = 0.559). Although neither stratified estimate reached statistical significance, the direction of the effect (lower post-treatment Hb in females) was consistent across both periods, supporting the robustness of the overall finding that female sex is an independent risk factor for lower post-treatment Hb in the full cohort.

### 3.6 Hepatotoxicity has no clear association with vancomycin AUC

Hepatotoxicity had no significant association with vancomycin AUC. Although the 1 g q12 h and 0.5 g q8 h regimens led to significant decreases in TBIL (P = 0.001 and P = 0.008, respectively; Table 7), the 0.5 g q8 h regimen led to increases in alkaline phosphatase (ALP) and gamma-glutamyl transferase (r-GT). Neither C_trough_ nor AUC had significant correlations with post-treatment TBIL (Table 6). ROC analysis showed that C_trough_ (AUROC = 0.48, Figure 6b) and AUC (AUROC = 0.47, Figure 6e) had no predictive value for hepatotoxicity. Multivariate analysis demonstrated that higher body weight was independently associated with a higher post-treatment TBIL (β = 0.492, P < 0.001; Table 5), possibly reflecting underlying conditions such as non-alcoholic fatty liver disease.

## 4 Discussion

We performed a retrospective cohort study of 148 patients and 193 regimens and evaluated clinical efficacy in 59 culture-confirmed Gram-positive infections. Our analysis of PK/PD targets and clinical success was limited to the 26 patients with staphylococcal infections who received a 1 g q12 h regimen; within this subgroup, traditional PK/PD targets including C_trough_, AUC, and AUC/MIC had no significant association with clinical success. However, comprehensive analysis of the full TDM-guided cohort may provide several key insights that could aid in optimizing vancomycin dosing regimens. First, we identified dosing frequency as an independent and modifiable factor that significantly influences C_trough_ without altering the overall daily exposure (AUC_0–24_). Second, we confirmed that AUC was a better predictor of nephrotoxicity than C_trough_, consistent with current guidelines. Finally, we identified female gender as a significant and independent risk factor for vancomycin-associated anemia, highlighting the need for gender-aware monitoring strategies.

### 4.1 PK/PD targets and real-world outcomes

The lack of correlation between clinical efficacy and traditional PK/PD targets (e.g., AUC/MIC ≥400) in our subgroup of patients with staphylococcal infections is consistent with findings from recent large-scale studies (Tsutsuura et al., 2021; Lodise et al., 2020; Tongsai and Koomanachai, 2016; Wan et al., 2018). Although the pharmacodynamic activity of vancomycin was evident based on decreased body temperature, WBCs, neutrophils, and CRP (Table 3), rigidly targeting a specific PK parameter seemed to provide limited additional benefit in our real-world setting. Furthermore, the inherent variability in MIC testing, particularly when using the twofold dilution method, makes the AUC/MIC an unreliable target, this has led expert guidelines to recommend focusing on a uniform AUC target (400–600 mg⋅h/L) regardless of the exact MIC value (M et al., 2022). Our findings do not negate the importance of achieving an adequate AUC, but emphasize that TDM should be viewed as one component of a comprehensive clinical assessment, not a standalone guarantor of efficacy. Because the subgroup analysis (22 successes *vs.* 4 failures) was underpowered (*post hoc* power: 19.3% for AUC; 50.8% for C_trough_), the lack of significance does not demonstrate that PK/PD targets are irrelevant, and the null findings should be interpreted as hypothesis-generating rather than conclusive.

### 4.2 Dosing frequency–a hypothesis-generating observation

Our multivariate analysis (Table 5) demonstrated that dosing frequency independently affected C_trough_ without altering the total daily AUC. We acknowledge that higher trough concentrations with more frequent dosing (e.g., q4 h) are pharmacokinetically expected. The key observation is that this increase in C_trough_ occurred without altering total daily AUC, which supports the PK/PD rationale that fractionated regimens can achieve rapid target attainment without increasing total drug exposure or concentration-dependent nephrotoxicity. This finding should be viewed as a hypothesis-generating pharmacokinetic observation rather than an unexpected discovery, and it requires prospective clinical validation. Regimens with more frequent dosing, such as 0.5 g q4h, achieved significantly higher trough concentrations than regimens with less frequent dosing that had the same total daily dose. This suggests that more frequent dosing may provide an advantage for rapid attainment of a target concentration in severe infections, infections caused by pathogens with higher MICs, or patients with augmented renal clearance (CrCl ≥130 mL/min). Although the post-antibiotic effect suggests that dosing intervals shorter than 8 h may not further enhance bacterial killing theoretically, our findings indicate that more frequent dosing can effectively increase trough concentration and improve bacterial killing in clinical practice, consistent with previous case studies (Zhang et al., 2021). Clinically, this rapid elevation of the trough concentration is particularly valuable in critically ill patients. Compared with continuous infusion, intermittent infusion offers greater convenience and flexibility. However, given the higher trough levels, careful AUC-guided monitoring of nephrotoxicity is warranted.

Vancomycin exhibits both time- and concentration-dependent pharmacodynamics, and efficacy is primarily driven by the AUC/MIC ratio (Freitas et al., 2020). Therefore, optimizing dosing frequency can increase T > MIC without increasing peak concentrations or total daily AUC. Although more frequent dosing leads to higher trough concentrations, our data indicate that AUC, rather than trough concentration, is the primary driver of nephrotoxicity. Thus, with careful AUC-guided monitoring, this strategy can enhance bacterial killing without increasing the concentration-dependent nephrotoxicity that is associated with dose escalation (Lodise et al., 2008). This PK/PD-guided approach, which we previously explored in case studies (Zhang et al., 2021), represents a shift from focusing solely on total daily dose to prioritizing dose frequency to achieve a more favorable risk-benefit profile. Nevertheless, our findings require prospective validation.

### 4.3 AUC-guided monitoring for renal safety

In the 167 TDM-guided regimens used to analyze the primary nephrotoxicity endpoint of post-treatment CrCl below 50 mL/min, the C_trough_ and the AUC had predictive value, but the AUC had superior discriminability (AUROC: 0.754 *vs.* 0.659). To further validate the robustness of this finding, we conducted a sensitivity analysis defining nephrotoxicity as a ≥30% decline in CrCl from baseline. Consistently, AUC remained superior to C_trough_ in predicting this endpoint, with an AUROC of 0.629 (95% CI: 0.396–0.863) *versus* 0.615 (95% CI: 0.426–0.804) for C_trough_. These findings aligns with a growing body of evidence. For example, meta-analyses by Tsutsuura et al. and Aljefri et al. reported a strong association between high vancomycin AUC (typically >600–650 μg.h/mL) and increased risk of AKI, and demonstrated that AUC-guided monitoring significantly reduces nephrotoxicity compared with C_trough_-guided monitoring (Aljefri et al., 2019; Tsutsuura et al., 2021; Abdelmessih et al., 2022). Experimental studies showed that increases in urinary biomarkers of kidney injury, such as kidney injury molecule-1, correlated more strongly with AUC than with C_trough_ (Avedissian et al., 2019). Our findings, which used a Bayesian method with JPKD software to estimate AUC, are consistent with current consensus guidelines from the IDSA and the Japanese Society of Chemotherapy, which recommend using Bayesian-informed AUC-guided dosing to personalize vancomycin therapy and increase renal safety (Rybak et al., 2020; M et al., 2022).

We acknowledge a potential mathematical artifact inherent in the Cockcroft-Gault equation, which contains serum creatinine in the denominator; for a given absolute increase in serum creatinine, the relative decline in calculated CrCl is larger when baseline serum creatinine is low. However, our primary endpoint used an absolute threshold (post-treatment CrCl <50 mL/min), which partially mitigates this issue. Furthermore, the sensitivity analysis based on relative decline, although more susceptible to this mathematical property, still confirmed the robustness of AUC’s predictive superiority. In our multivariate model, pre-treatment renal function emerged as the primary predictor of post-treatment CrCl, which likely reflects the strong collinearity between AUC and baseline renal function, as well as the limited number of nephrotoxicity events. Nonetheless, the strong association between a high AUC and nephrotoxicity underscores the importance of early monitoring of AUC to identify at-risk patients and guide dose adjustments (Aljefri et al., 2019; Tsutsuura et al., 2021; Avedissian et al., 2019; Ueda et al., 2022; Pais et al., 2019).

### 4.4 Gender-specific toxicity

The baseline hemoglobin level was lower in females (median 101 g/L, IQR 89–112) than in males (116 g/L, IQR 100–132). The median post-treatment Hb level in females was 98 g/L (IQR 84.5–106) and 109.50 g/L (IQR 94.75–125)in males, with a median decline of 3 g/L and 6.5 g/L, respectively. Multivariate analysis (Table 5) confirmed that female gender was an independent risk factor for a lower post-treatment level of Hb (β = 10.735, *P* = 0.002), after adjusting for baseline Hb, body weight, AUC, C_trough_, and daily dose. The lower baseline Hb in women may partly contribute to this finding, but the independent effect of gender suggests an additional susceptibility. Of note, when we compared the raw change in hemoglobin (ΔHb) between males and females without adjustment, the difference did not reach statistical significance (Table 2). However, after accounting for baseline Hb and other covariates in the multivariate model, female sex remained a significant predictor of lower post-treatment Hb. This discrepancy underscores the importance of adjusting for baseline physiological differences; the univariate comparison of ΔHb was confounded by the lower starting Hb level in females, whereas the multivariate analysis isolated the true effect**.** This independent sex effect highlights the need for gender-aware therapeutic monitoring; clinicians should be particularly vigilant for declining Hb levels in females receiving vancomycin, especially those with low baseline Hb or those receiving higher-intensity or prolonged regimens. Notably, the weak predictive value of AUC and C_trough_ for anemia in our ROC analysis (AUROC 0.62–0.63) suggests that the increased risk in women may be idiosyncratic or related to patient-specific factors rather than drug exposure metrics.

This study has several limitations. First, as a single-center retrospective study, the effect of unmeasured confounders (e.g., illness severity, source control and concomitant medications that may affect hemoglobin levels (e.g., β-lactam antibiotics)) cannot be fully excluded. Second, the sample size was insufficient for robust subgroup analyses. This was particularly true for the comparison of clinical success and failure in the subgroup which received the 1 g q12 h regimen (n = 26, with 4 failures), which is underpowered to detect meaningful differences. Third, toxicity was defined by laboratory thresholds rather than clinical events. Fourth, real-world deviations in dosing schedules and trough sampling times may have introduced measurement errors. Fifth, variations in renal function during vancomycin therapy could not be fully captured from pre- and post-treatment measurements, and there could have been bias in the estimates of nephrotoxicity risk. Sixth, clinical efficacy was assessed only in culture-confirmed cases (59/148 patients, 39.9%), while the majority of the cohort had suspected infections without microbiological documentation. This design choice was necessary to avoid misclassification of clinical response in patients without confirmed Gram-positive infections, but it may limit the generalizability of our efficacy findings and introduce selection bias. The retrospective nature of the study precluded routine culture in all patients, which reflects real-world clinical practice but should be considered when interpreting the efficacy results. Seventh, given its retrospective design, limited subgroup sample sizes, and the *post hoc* nature of the sex-anemia finding, our results are exploratory and hypothesis-generating, and warrant prospective validation. Finally, our findings on dosing frequency are observational and require prospective validation because we could not assess microbiologic cure (repeat negative cultures). Follow-up cultures were not routinely obtained for non-bacteremic infections (e.g., pneumonia, skin/soft tissue infections).

## 5 Conclusion

Optimizing vancomycin therapy requires a multi-dimensional approach that balances efficacy and safety. In the small staphylococcal subgroup of this study, traditional PK/PD targets did not correlate with clinical success, however, given the underpowered analysis (n = 26, with 4 failures), this must be considered as a hypothesis-generating finding, not a definitive result. In the full cohort (n = 175), dosing frequency independently affected C_trough_ without altering AUC, an observation that does not indicate efficacy and requires prospective validation. AUC values were superior to C_trough_ values for predicting nephrotoxicity, and female gender was a risk factor for vancomycin-associated anemia. Personalized regimens that integrate optimized dosing frequency, monitoring of AUC, and gender-aware assessments may improve the balance of efficacy and safety in vancomycin regimens, but require confirmation in future studies.

## Data Availability

The raw data supporting the conclusions of this article will be made available by the authors, without undue reservation.
