# Supplementary material for: Optimizing vancomycin therapy: the critical role of dosing frequency, AUC monitoring and gender: a retrospective cohort study
Source: Front Pharmacol. 2026 Aug 19;17:1843465. doi: 10.3389/fphar.2026.1843465 (PMC13533476; doi:10.3389/fphar.2026.1843465)
Supplement: Supplementary file 1 [file Supplementaryfile1.docx]

**Supplementary Table S1. Concomitant use of potential nephrotoxic and hematotoxic agents during vancomycin therapy (n = 148).***

| **Concomitant agent category** | **Patients (n), %** |  |
| --- | --- | --- |
| **Any nephrotoxic agent** |  |  |
| NSAIDs | 95, 64.2% |  |
| Contrast media | 16, 10.8% |  |
| Aminoglycosides | 29, 19.6% |  |
| Amphotericin B | 0, 0% |  |
| Loop diuretics | 37, 25% |  |
| **Any hematotoxic agent** | 10, 6.8% |  |

*Percentages are calculated based on the total cohort (N = 148).

Due to the small numbers of events and incomplete documentation (e.g., fluid boluses), these variables were not included in multivariate models.

**Supplementary Table S2. Comparison of baseline characteristics and main outcomes between pre-pandemic and pandemic cohorts (n=175 regimens)**

| Characteristic | Pre-pandemic (n=102) | Pandemic (n=73) | Z/ χ² | P value |
| --- | --- | --- | --- | --- |
| **Baseline characteristics, median (Q1, Q3)** |  |  |  |  |
| Age (years) | 60.5 (47.0, 68.5) | 53.0 (47.0, 68.0) | −0.664 | 0.506 |
| Body weight (kg) | 61.5.0 (54.0, 72.0) | 60.0 (51.8, 70.5) | −0.848 | 0.396 |
| Treatment duration (days) | 9.0 (6.0, 14.3) | 9.0 (5.5, 14.0) | −0.573 | 0.566 |
| Daily dose (g) | 2.0 (2.0, 2.0) | 2.0 (2.0, 2.0) | -0.718 | 0.472 |
| AUC (µg·h/mL) | 391.9 (321.0, 491.6) | 448.0 (362.4, 550.1) | -2.077 | 0.038 |
| C_trough_ (µg/mL) | 11.0 (8.5, 15.9) | 13.5 (10.4, 18.5) | -1.917 | 0.055 |
| Tmax (℃) | 38.7 (37.6, 39.5) | 37.6 (36.6, 39.1) | -1.698 | 0.090 |
| WBC(×10⁹/L) | 9.1(7.9, 12.9) | 11.8 (5.7, 16.0) | -1.008 | 0.313 |
| N% (%) | 76.6(70.5, 83.8) | 81.4 (73.8, 86.0) | -0.660 | 0.510 |
| CRP (mg/L) | 119.7 (69.4, 170.4) | 44.7 (20.2, 135.2) | -1.291 | 0.197 |
| PCT (ng/mL) | 1.4 (0.2, 4.2) | 0.3 (0.1, 0.6) | -1.408 | 0.159 |
| RBC (×10¹²/L) | 3.8 (3.4, 4.4) | 3.5 (3.0, 4.0) | -2.840 | 0.005 |
| Hb (g/L) | 115.5 (99.5, 131.8) | 103.5 (88.0, 117.5) | -3.250 | 0.001 |
| PLT (×10⁹/L) | 288.0 (189.5, 370.8) | 255.0 (185.3, 358.5) | -0.633 | 0.527 |
| ALT  (U/L) | 36.0 (18.0, 57.0) | 22.0 (15.0, 51.0) | -1.749 | 0.080 |
| AST  (U/L) | 29.0 (20.0, 46.0) | 28.0 (18.0, 43.0) | -0.259 | 0.795 |
| ALP (U/L) | 90.0 (74.0, 135.0) | 81.0 (58.0, 113.0) | -2.253 | 0.024 |
| r-GT (U/L) | 61.0 (28.0, 110.0) | 40.0 (25.0, 74.0) | -1.795 | 0.073 |
| TBIL (µmol/L) | 8.4 (6.1, 15.7) | 10.3 (7.2, 13.8) | -0.879 | 0.379 |
| DBIL (µmol/L) | 3.0 (1.8, 4.7) | 3.2 (1.9, 6.1) | -0.679 | 0.497 |
| Scr (µmol/L) | 62.0 (52.0, 70.0) | 63.0 (50.0, 71.5) | -0.008 | 0.994 |
| CrCl (mL/min) | 100.3 (78.5, 124.7) | 91.8 (71.2, 129.4) | -0.761 | 0.447 |
| BUN (mmol/L) | 4.8 (3.5, 6.4) | 4.9 (3.5, 6.9) | -0.252 | 0.801 |
| **Categorical variables, n (%)** |  |  |  |  |
| Male sex | 76 (74.5) | 39 (53.4) | 8.396 | 0.004 |
| BSI (vs. non-BSI) | 16 (61.5) | 15 (53.6) | 0.350 | 0.554 |
| ICU (vs. non-ICU) | 10 (9.8) | 19 (26.0) | 8.100 | 0.004 |

**Table footnotes:**

Abbreviations: AUC, area under the curve; C_trough_, trough concentration; Tmax, maximal body temperature; WBC, white blood cell count; N%, neutrophil percentage; CRP, C‑reactive protein; PCT, procalcitonin; RBC, red blood cell count; Hb, hemoglobin; PLT, platelets; ALT, alanine aminotransferase; AST, aspartate aminotransferase; ALP, alkaline phosphatase; r‑GT, gamma‑glutamyl transferase; TBIL, total bilirubin; DBIL, direct bilirubin; Scr, serum creatinine; CrCl, creatinine clearance; BUN, blood urea nitrogen; ICU, intensive care unit; BSI, bloodstream infection; Pre‑pandemic, patients treated from 2013 to 2019; Pandemic, patients treated from 2020 to 2021.

Continuous variables presented as median (Q1, Q3), compared using Mann–Whitney U test (Z values). Categorical variables presented as n (%), compared using Pearson chi‑square test (χ² values). This table compares baseline characteristics and key pharmacokinetic parameters between the two periods based on the full TDM‑guided cohort (n=175 regimens). Efficacy‑related stratified analysis (sex‑Hb association) was performed in 54 treatment regimens. These 54 regimens were derived from the intersection of the 175 TDM‑guided regimens and the 59 culture‑confirmed regimens in the overall cohort (n=193). The remaining 5 of the 59 culture‑confirmed cases were excluded from this stratified analysis because they lacked therapeutic drug monitoring (TDM) data.

**Supplementary Table S3. Distribution of staphylococcal isolates in the 26 patients receiving the 1g q12h regimen, stratified by clinical outcome**

| **Organism Type** | **Total (n=26) n (%)** | **Clinical Success (n=22) n (%)** | **Clinical Failure (n=4) n (%)** |
| --- | --- | --- | --- |
| **S. aureus (combined)** | 9 (34.6%) | 7 (31.8%) | 2 (50.0%) |
| ─ MRSA (oxacillin-resistant) | 6 (23.1%) | 5 (22.7%) | 1 (25.0%) |
| ─ MSSA (oxacillin-susceptible) | 3 (11.5%) | 2 (9.1%) | 1 (25.0%) |
| **CoNS (combined)** | 19 (73.1%) | 16 (72.7%) | 3 (75.0%) |
| ─ S. hominis | 8 (30.8%) | 7 (31.8%) | 1 (25.0%) |
| ─ S. epidermidis | 7 (26.9%) | 6 (27.3%) | 1 (25.0%) |
| ─ S. haemolyticus | 3 (11.5%) | 3 (13.6%) | 0 (0%) |
| ─ S. warneri | 1 (3.8%) | 1 (4.5%) | 0 (0%) |

Footnote: *One patient had polymicrobial infection with both S. aureus (MSSA) and CoNS (S. epidermidis and S. hominis); therefore, the number of isolates (28) exceeds the number of patients (26), and percentages calculated with 26 patients as the denominator do not sum to 100%. Abbreviations: MRSA, methicillin-resistant Staphylococcus aureus; MSSA, methicillin-susceptible Staphylococcus aureus; CoNS, coagulase-negative staphylococci.

**Supplementary Table S4. Concomitant use of anti-staphylococcal antibiotics in the 26 patients with staphylococcal infections receiving the 1g q12h regimen, stratified by clinical outcome**

| **Concomitant Antibiotic Category** | **Total (n = 26) n (%)** | **Clinical Success (n = 22) n (%)** | **Clinical Failure (n = 4) n (%)** |
| --- | --- | --- | --- |
| **Any concomitant systemic antibiotic** | 22 (84.6%) | 18 (81.8%) | 4 (100%) |
| **Anti-MSSA β-lactams** (cefazolin, cloxacillin, oxacillin, nafcillin) | 0 (0%) | 0 (0%) | 0 (0%) |
| **Anti-MRSA agents** (rifampin, rifamycin; linezolid/daptomycin/tigecycline not used) | 2 (7.7%) | 1 (4.5%) | 1 (25.0%) |
| Rifampin | 1 (3.8%) | 1 (4.5%) | 0 (0%) |
| Rifamycin | 1 (3.8%) | 0 (0%) | 1 (25.0%) |
| **Other anti-Gram-positive antibiotics** (moxifloxacin, fosfomycin, levofloxacin) | 8 (30.8%) | 8 (36.4%) | 0 (0%) |
| ─ Moxifloxacin | 5 ( 19.2%) | 5(22.7%) | 0 (0%) |
| ─ Fosfomycin | 2 (7.7%) | 2 (9.1%) | 0 (0%) |
| ─ Levofloxacin | 1 (3.8%) | 1 (4.5%) | 0 (0%) |
| **Broad-spectrum antibiotics with weak anti-staphylococcal activity** | 17 (65.4%) | 14 (63.6%) | 3 ( 75.0%) |
| ─ Cefoperazone/sulbactam | 9 (34.6%) | 7 (31.8%) | 2 (50%) |
| Meropenem | 2 (7.7%) | 2 (9.1%) | 0(0%) |
| Piperacillin/sulbactam | 3 ( 11.5%) | 2 (9.1%) | 1 (25.0%) |
| Imipenem/cilastatin | 2 (7.7%) | 2 (9.1%) | 0 (0%) |
| Amikacin | 3 ( 11.5%) | 2 (9.1%) | 1 (25.0%) |
| Ceftriaxone | 1 (3.8%) | 0 (0%) | 1 (25.0%) |
| Gentamicin | 1 (3.8%) | 1 (4.5%) | 0 (0%) |
| Ceftazidime | 1 (3.8%) | 1 (4.5%) | 0 (0%) |

**Abbreviations:** MSSA, methicillin-susceptible Staphylococcus aureus; MRSA, methicillin-resistant Staphylococcus aureus.

**Footnote:** Percentages are calculated using the number of patients (n = 26) as the denominator. The sum of subcategory frequencies exceeds the category total because some patients received multiple antibiotics within the same category; the category total represents the number of unique patients who received at least one antibiotic in that category. Notably, no patient received anti-MSSA β-lactams (cefazolin, cloxacillin, oxacillin, or nafcillin) or other anti-MRSA agents (linezolid, daptomycin, or tigecycline) during vancomycin therapy; only rifampin and rifamycin were used as concomitant anti-MRSA agents in 2 patients.

**Supplementary Table S5. Microbiologic follow‑up in patients with confirmed Gram‑positive infections (n = 59).**

| Variable | n (%) |
| --- | --- |
| **All confirmed Gram‑positive infections** | 59 (100) |
| ─ Repeat cultures obtained | 26(44.1) |
| ─ Negative follow‑up culture | 66 |
| ─ Persistent positive culture | 12 |
| **Bacteremic infections** | 22 (37.3) |
| ─ Repeat blood cultures obtained | 18(81.8) |
| ─ Negative follow‑up culture | 43 |
| ─ Persistent positive culture | 4 |
| **Non‑bacteremic infections** | 37 (62.7) |
| ─ Repeat cultures obtained* | 8(21.6) |
| ─ Negative follow‑up culture | 23 |
| ─ Persistent positive culture | 8 |

* Repeat cultures were not routinely performed for non‑bacteremic infections unless clinically indicated; therefore, the denominator for follow‑up in this subgroup is limited.
